# Head turning is an effective cue for gaze following: Evidence from newly sighted individuals, school children and adults

**DOI:** 10.1016/j.neuropsychologia.2022.108330

**Published:** 2022-07-14

**Authors:** Paula Rubio-Fernandez, Vishakha Shukla, Vrinda Bhatia, Shlomit Ben-Ami, Pawan Sinha

**Affiliations:** a University of Oslo, Norway; b Massachusetts Institute of Technology, USA; c New York University, USA; d Jawaharlal Nehru University, India; e Tel Aviv University, Israel

**Keywords:** Gaze following, Referential intent, Theory of mind, Motion cues, Congenital cataracts, Developmental pathways

## Abstract

In referential communication, gaze is often interpreted as a social cue that facilitates comprehension and enables word learning. Here we investigated the degree to which head turning facilitates gaze following. We presented participants with static pictures of a man looking at a target object in a first and third block of trials (pre- and post-intervention), while they saw short videos of the same man turning towards the target in the second block of trials (intervention). In Experiment 1, newly sighted individuals (treated for congenital cataracts; N = 8) benefited from the motion cues, both when comparing their initial performance with static gaze cues to their performance with dynamic head turning, and their performance with static cues before and after the videos. In Experiment 2, neurotypical school children (ages 5–10 years; N = 90) and adults (N = 30) also revealed improved performance with motion cues, although most participants had started to follow the static gaze cues before they saw the videos. Our results confirm that head turning is an effective social cue when interpreting new words, offering new insights for a pathways approach to development.

Developmental research has investigated gaze following – the ability to look where another person is looking, as an early precursor of Theory of Mind – the human capacity to understand mental states (Wellman, 2014). In this view, gaze following is tightly connected to the development of joint attention: the human social ability to share one’s attention with others (Mundy and Newell, 2007). Gaze following is of fundamental importance in verbal interaction because of its referential nature: people often display emotions and talk about the physical world around them, so monitoring their line of gaze offers a cue to their referential intent. Gaze following also supports word learning, giving young children a cue to map verbal labels onto specific objects (Baldwin, 1995; Tomasello, 1995; Bloom, 2002). Here we investigated how gaze following supports referential communication in a unique population: newly sighted children who were treated for dense congenital cataracts after several years of visual deprivation. Adopting the name of the humanitarian/scientific organization that sought their treatment in India (Sinha and Held, 2012; Sinha, 2013), we will refer to these children as *Prakash children*.

Several studies with newborns and young infants have demonstrated their ability to discriminate human facial features, in particular the eyes (Farroni et al., 2002, 2005). Hood et al. (1998) showed that 3-month-old infants can discriminate gaze direction on an adult face and shift their own attention accordingly. More importantly for the purpose of our study, Farroni et al. (2000) replicated this finding with 4-month-old babies, and discovered that infants were cued by the *motion* of the adult’s pupils: when the pupils of the stimulus face stayed still while the face was displaced, infants were cued by the direction of the face rather than by the eye gaze (which appeared to shift in the opposite direction).

Interactive studies with babies have also shown that as early as 3 months, they can direct their attention in the direction of an adult’s head turn (Scaife and Bruner, 1975; D’Entremont et al., 1997). However, some of these early studies observed that until 18 months of age, infants relied on the adult’s head motion to orient their own attention, suggesting that they may not be sensitive to the adult’s gaze *per se* (Lempers, 1979; Butterworth and Jarrett, 1991; Moore et al., 1997; Corkum and Moore, 1995, 1998; for a computational model, see Triesch et al., 2006). Brooks, Meltzoff and colleagues challenged this lean interpretation of infants’ gaze behavior with a novel experimental paradigm that kept the adult’s head motion constant, but varied whether the adult had their eyes open or closed (Caron et al., 2002a, 2002b; Brooks and Meltzoff, 2002; Meltzoff and Brooks, 2007). Under these conditions, 10–12-month-old infants started to show gaze following, turning to look at a target object only when the adult had their eyes open.

All the above studies suggest that directed motion perception is an important cue for gaze following. Whether with larger head turns, or more subtle pupil shifts, infants reorient their attention cued by their perception of motion in an adult’s face. This is a key finding for the present study, as it motivated our main research question: when Prakash children gain vision later in childhood, do they also rely on motion perception to follow an adult’s gaze? Before addressing this question, it is worth looking at the connection between gaze following and another fundamental capacity in human development: learning new words.

## 1. The connection between gaze following and word learning

Brooks and Meltzoff (2005) observed a strong positive correlation between gaze-following behavior at 10–11 months and subsequent language scores at 18 months (see also Carpenter et al., 1998; Morales et al., 2000; Mundy et al., 2007; Pons et al., 2019). In the context of language acquisition, gaze has been treated as a *social cue*: an ostensive signal of referential intent that assists infants and children in drawing inferences about new words in their everyday interactions (Baldwin and Moses, 2001; Csibra and Gergely, 2009). The standard paradigm in this literature compares a follow-in condition, where an adult labels an object that the child is already attending to, with a discrepant condition, where the child needs to shift their own attention to adopt the adult’s perspective. Early studies have shown that before 2 years, children are above chance only in the follow-in condition. However, from 24 months onward, children show learning in both conditions, also revealing more *contingent looking*: coordinated attention between the speaker and the intended object (Baldwin, 1993; Baldwin et al., 1996; Moore et al., 1999; Hollich et al., 2000).

In a recent study, Bang and Nadig (2020) showed that children use the directional information provided by both gaze and an arrow to learn label-referent mappings. Critically, however, children attended differently to the social cue and the arrow: children looked longer at the target object in the gaze condition and revealed more contingent looking between the speaker and the referent than between the arrow and referent. The authors interpreted these results in line with theoretical accounts of gaze as a social cue that signals referential intent (Baldwin, 1993; Baldwin and Moses, 2001; Csibra and Gergely, 2009). However, it is important to note that word learning also requires pragmatics, and not only gaze following (see Nurmsoo and Bloom, 2008). In a study with children at risk of autism, Gliga et al. (2012) showed that gaze was necessary but not sufficient for successful word learning: those children showing poor social skills revealed unimpaired gaze following but limited new word acquisition. Gliga et al. argued that ASD children may be sensitive to socio-pragmatic cues but lack the ability to reliably deploy them in communication.

The Prakash children investigated in this study have shown good pragmatic performance in referential communication, providing sufficiently informative descriptions of referents (e.g., ‘I prefer the small cup’ in the context of two cups). While comparable to neurotypical controls in their pragmatic ability to preempt ambiguity, these newly sighted children paid less attention to their interlocutor’s face. Recordings from eye-tracking glasses revealed that Prakash children made fewer fixations to their interlocutor’s face than their controls, both as speakers and listeners (Rubio-Fernandez et al., in preparation). Given their good pragmatic skills but lesser interest in faces, the present study investigated whether and how Prakash children use gaze following in the context of word learning.

## 2. The importance of motion cues for children with delayed sight onset

Vision scientists have long recognized that individuals who acquire sight late in life provide a unique window into many important aspects of visual development (Valvo, 1971). Given the importance of gaze information in learning new words and establishing common ground (Baldwin and Moses, 2001; Csibra and Gergely, 2009), the same could be argued about language acquisition and pragmatic development. In a recent study comparing 23 children treated for congenital cataracts with 57 neurotypical controls, McKyton et al. (2017) observed that the clinical group did not reveal a *gaze compatibility effect* in a standard gaze cueing paradigm. That is, children with late vision onset were not faster to touch a balloon on either side of a computer screen if they had been previously exposed to a face of a man looking in the same direction, relative to a condition where the man was looking in the opposite direction to the upcoming balloon. By contrast, the control group revealed a significantly stronger gaze compatibility effect, even if they were presented with blurred stimuli to match the reduced visual acuity of the clinical group. It is important to note, however, that the children with delayed sight onset had enough visual acuity to detect the eyes on the man’s face, but did not reveal automatic joint attention. The authors therefore concluded that the development of joint attention may be subject to critical periods, or at least be slow to develop after an extended period of blindness.

A possible reason why the newly sighted children in the study by McKyton et al. (2017) did not reveal automatic joint attention is that the gaze cue was static, rather than dynamic, and earlier studies with this population have confirmed the importance of motion cues for their visual development. Ostrovsky et al. (2009) tested three Prakash individuals (ages: 12, 13, 29) and provided longitudinal evidence that the early stages of visual parsing are characterized by overfragmentation of images, compromising object recognition as a result. However, their study showed that motion information effectively mitigates these difficulties: motion cues enabled integrating object features and facilitated the development of object representations that permitted recognition in static images.

For instance, when displaying the overlapping outlines of a square and a circle, Prakash participants would identify three shapes (by isolating the overlapping and non-overlapping areas), but when the square momentarily moved on the screen, participants were able to identify the two shapes correctly (even though the outlines continued to overlap during motion; for video footage of this test, see Sinha, 2009). Most importantly, Ostrovsky et al. (2009) reported that, following 10–18 months of visual experience, the Prakash individuals’ performance improved, being able to use previously ineffective cues to correctly parse many static scenes. These researchers concluded that motion information plays a fundamental role in organizing early visual experience (for evidence with infants, see Johnson, 2001) and that parsing skills can be acquired even after years of visual deprivation.

Studies of face processing have shown that dynamic and static cues may have different developmental trajectories and play differential roles in bootstrapping face processing mechanisms (Mondloch et al., 2003; Blais et al., 2017). More recent work from Project Prakash has also shown that motion cues facilitate facial expression recognition in children with delayed sight onset (Gilad-Gutnick et al., 2019). Here we investigated the degree to which motion cues facilitate Prakash children’s use of gaze following in referential communication. We used head turns as motion cues, instead of pupil shifts, because while Prakash children understand the role of eyes in visual perception (unlike young infants), they may not always have sufficient visual acuity to appreciate an adult’s gaze direction.

In an earlier case study with a 32-year-old woman who had been born blind and was treated from dense congenital cataracts at age 12, Ostrovsky et al. (2006) observed that she was at ceiling when detecting gaze direction, but based her judgements on head orientation rather than pupil position. Some of the Prakash children who participated in the present study had also performed a control task for another study, in which we obtained a baseline measure of gaze following by asking them to indicate to which side (right or left) another person was looking (Rubio-Fernandez et al., in preparation). On average, the Prakash children in that study were at chance when detecting gaze direction, which reaffirmed our decision to use head turns as motion cues (instead of pupil shifts) in the present study.

## 3. Weak and strong directionality cues

**Fig. 1.**
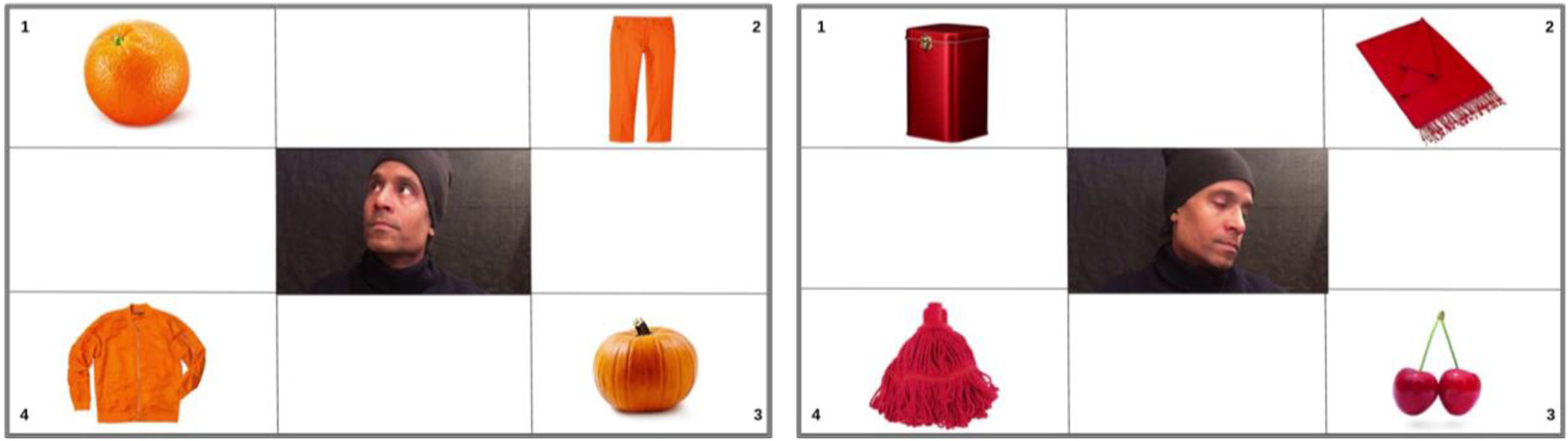
Sample displays from pre- and post-intervention trials (Blocks 1 and 3). The man’s face orientation corresponds with the end of the videos shown in intervention trials (Block 2).

Word learning studies have relied on a variety of measures including word recognition, referent selection and semantic descriptions (Bang and Nadig, 2020). Here we presented children with a referential communication task, in which they heard a male speaker name an object in an unfamiliar language. The target object was one amongst four objects of the same color, placed in the four corners of a visual display. In the center of the screen, children could also see the face of a man gazing towards the named object (see Fig. 1 for sample displays). Trials were presented in three blocks: the first and third blocks included static pictures of the man *gazing towards* the named object, while the middle block included videos of the same man *turning towards* the target object. The man did not utter the name of the target object in order to avoid that Prakash children would focus on the movement of the mouth, rather than the head turn (see Barenholtz et al., 2016; de Boisferon et al., 2018). This experimental set up therefore allowed us to investigate the extent to which referent selection improved in the dynamic video trials, relative to the static pictures shown in the first block. In addition, a comparison between the first and third blocks allowed us to measure whether the motion cues employed in the middle trials facilitated learning, revealing improved referent identification from static images in the last block of trials. In learning studies using this type of experimental design, the first and third blocks would be considered *pre-* and *post-intervention trials*, respectively, while the second block consists of *intervention trials*. We will also adopt this terminology when discussing our findings.

Our paradigm includes both *weak and strong directionality cues*. Thus, the static images of the man shown in pre- and post-intervention trials include several weak cues to his line of gaze: namely, pupil position, nose direction and face orientation. We consider those weak directionality cues because they are static. However, neurotypical children in the age ranges investigated here have had ample experience with both weak and strong directionality cues and have also been able to connect the two types of cues, allowing them to treat weak cues as reliable indicators of gaze direction. Prakash children, on the other hand, have not had such visual experiences and we therefore predict that they will need motion cues to appreciate the man’s line of gaze.

**Fig. 2.**
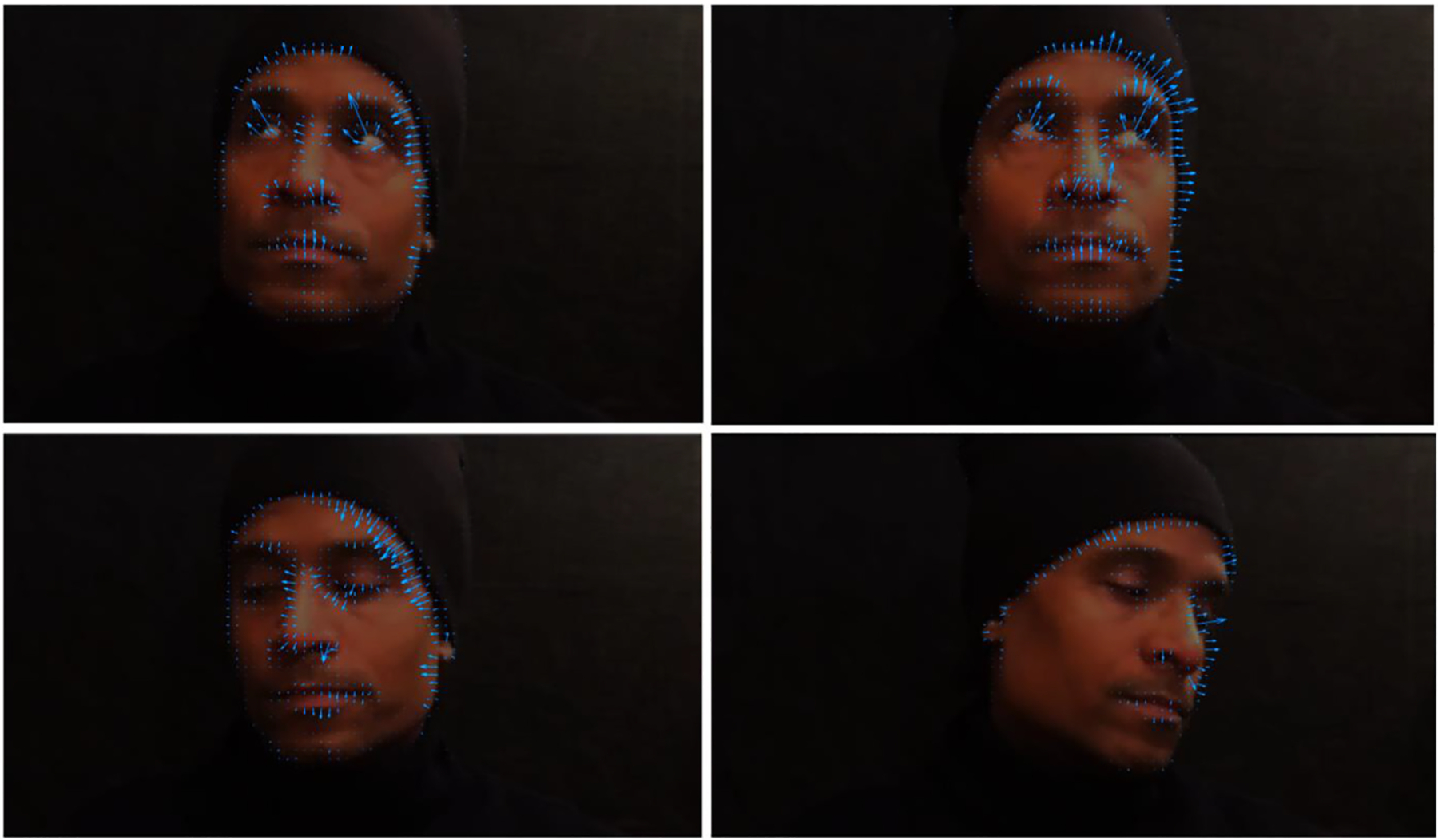
Optical flow estimations using the Horn-Schunck method (Horn and Schunck, 1981) with the four video sequences shown in the intervention trials (Block 2).

In order to confirm that the motion information in the intervention trials provided strong directionality cues for performing the task, we estimated optical flow in the stimulus videos using the Horn-Schunck method (Horn and Schunck, 1981). Unlike local methods for optic flow estimation, the Horn-Schunck approach is a ‘global’ method that employs regularization to enforce smoothness across the optic flow array. The results with our four video sequences show that the directions of the computed flow vectors provide a compelling cue for localizing the intended target (see Fig. 2). Note that no prior knowledge about faces or eyes is assumed in the calculation of optic flow; the flow vectors correspond simply to the displacements of image regions, irrespective of their semantics.

## 4. Experiment 1

### 4.1. Methods

#### 4.1.1. Participants

The study was approved by MIT’s IRB under protocol ‘Development of Visual Perception’ (\#:0403000050R016). For the first experiment, we recruited two groups of Prakash children and one control group.

##### 4.1.1.1. Prakash group.

At Dr. Shroff’s Charity Eye Hospital in Delhi (India), we tested five Prakash individuals (ages: 7, 13, 13, 15, 20; M = 13.2) at two test points. Participants P–B, P–C, P-D and P-E were first tested before surgery and then one month later, while P-A was tested only at the second test point because they did not have enough visual acuity to perform the task pre-op. Testing this first group of Prakash children allowed us to investigate the degree to which their gaze following improves in the first month after surgery. See Table 1 for demographic and clinical information.

All visual acuity measurements were collected using the Landodt’s C optotype of the Freiburg Visual Acuity Test (Bach, 1996) and run on a 17-inch Dell laptop. Measurements were collected from a 40 cm viewing distance and using both eyes. If subjects could not resolve any patterns from this viewing distance, the shortest distance from which they could correctly count fingers in 5/5 trials is reported (e.g., FC @ 30 cm in Table 2). In all other cases, acuity is reported in the Logarithm of the Minimum Angle of Resolution (logMAR) scale (Il and Je, 1976), where 0.0 logMAR is equivalent to 20/20 (normal vision) and 1.3 logMAR is equivalent to 20/400 (the legal threshold for blindness in the United States).

##### 4.1.1.2. Silver Linings group.

The second group of Prakash children consisted of three girls (ages: 10, 13, 18; M = 13.7), who were tested longitudinally at a boarding school in Delhi, where they are receiving individualized education. For this group, we will adopt the name of their boarding school, Silver Linings, but we want to highlight that these are also Prakash children who had received treatment for congenital cataracts earlier in their lives. Unlike the first group of Prakash children, these girls had received treatment one, two and ten years earlier. Our aim was therefore to investigate how much improvement in gaze following motion cues could afford them years after treatment. Participant SL-A showed worse performance in the first session and was therefore tested three times at 6-month intervals, while SL-B and SL-C were only tested twice, at the same time interval. See Table 2 for demographic and clinical information.

##### 4.1.1.3. Control group.

We recruited 9 neurotypical children in a primary school in Delhi (ages: 11–13; M = 12.0) to serve as a control group. We had two aims in testing this third group of children: we wanted to validate the task using eye tracking and to estimate whether control children’s responses would reveal enough variability to justify running a second experiment with neurotypical participants.

#### 4.1.2. Materials, task design and procedure

##### 4.1.2.1. Prakash group and controls.

A set of 48 slides with a white background were created showing 4 objects of the same color, one in each corner. In 32 of the slides, a picture with a dark background was placed in the center, showing a man looking towards one of the corners. The same 4 pictures of the man looking at one of the corners were used repeatedly in these 32 slides. These pictures were obtained from the last fame of the videos used in dynamic trials. The 16 remaining slides contained a short video of the same man turning his face towards the target object from a neutral, forward-looking position. The 32 slides with pictures were evenly distributed between trial blocks 1 and 3, counterbalancing the position of the target object and the direction of the man’s gaze (which always matched). The 16 slides with videos were presented in the second trial block (also counterbalanced). Each block included 16 trials.

The names of the target objects were recorded in either Spanish or Norwegian to avoid words that were phonologically similar to the Hindi name for the object (which would have provided an additional cue, since all participants in the study were Hindi speakers). Each trial started with a slide containing only the 4 objects, while the name of the target object was played once through speakers. These slides were displayed for 3000 ms. Each initial display was followed by the corresponding slide containing the objects plus the face of the man, either as a static image or as a short video, depending on the trial block. The follow-up slides with the face of the man were also displayed for 3000 ms (both static and video trials). The task was built using the Experiment Builder in a RED-m eye-tracking computer by SMI. Due to calibration problems with the Prakash group, eye movements were only recorded for the control group.^1^

Participants were told that they would see a series of displays with four objects and they would hear the name of one of the objects in a foreign language that they did not know. Then a picture of a man who speaks that language would appear in the center of the screen and will give the child a clue to find the object. The child was asked to follow the clue and choose the correct object by pointing at it. Children’s responses were recorded with an external camera for later coding. Children did not receive feedback during performance.

##### 4.1.2.2. Silver Linings group.

The same materials and task design that were used with the Prakash group and the controls were used again with the Silver Linings group. However, because of the COVID-19 pandemic, we were not able to test this group in person. In order to administer the task online, we streamlined the procedure by showing the face of the man at the same time as the name of the object was played (i.e. the objects/name were not shown prior to the man’s face but synchronously). In addition, the objects on each slide were numbered 1–4, clockwise, so that the children could respond by number. The task was built as one continuous video and paused at each trial for the participant to respond.

The Silver Linings group was tested over Zoom with the help of a school assistant. The experimenter shared their screen and recorded the session. The task began with 4 practice trials showing 4 color patches, one in each corner of the display. To familiarize the children with the numbering of the corners, the experimenter named a color in each practice trial and the participant was asked to identify the corresponding corner by number. We avoided using pictures of real objects in the practice trials in case the Silver Linings girls were not able to recognize the objects. However, the same instructions were used with this group of participants. The only difference was the form of the children’s response (pointing vs object number).

## 5. Results

### 5.1. Prakash group

**Fig. 3.**
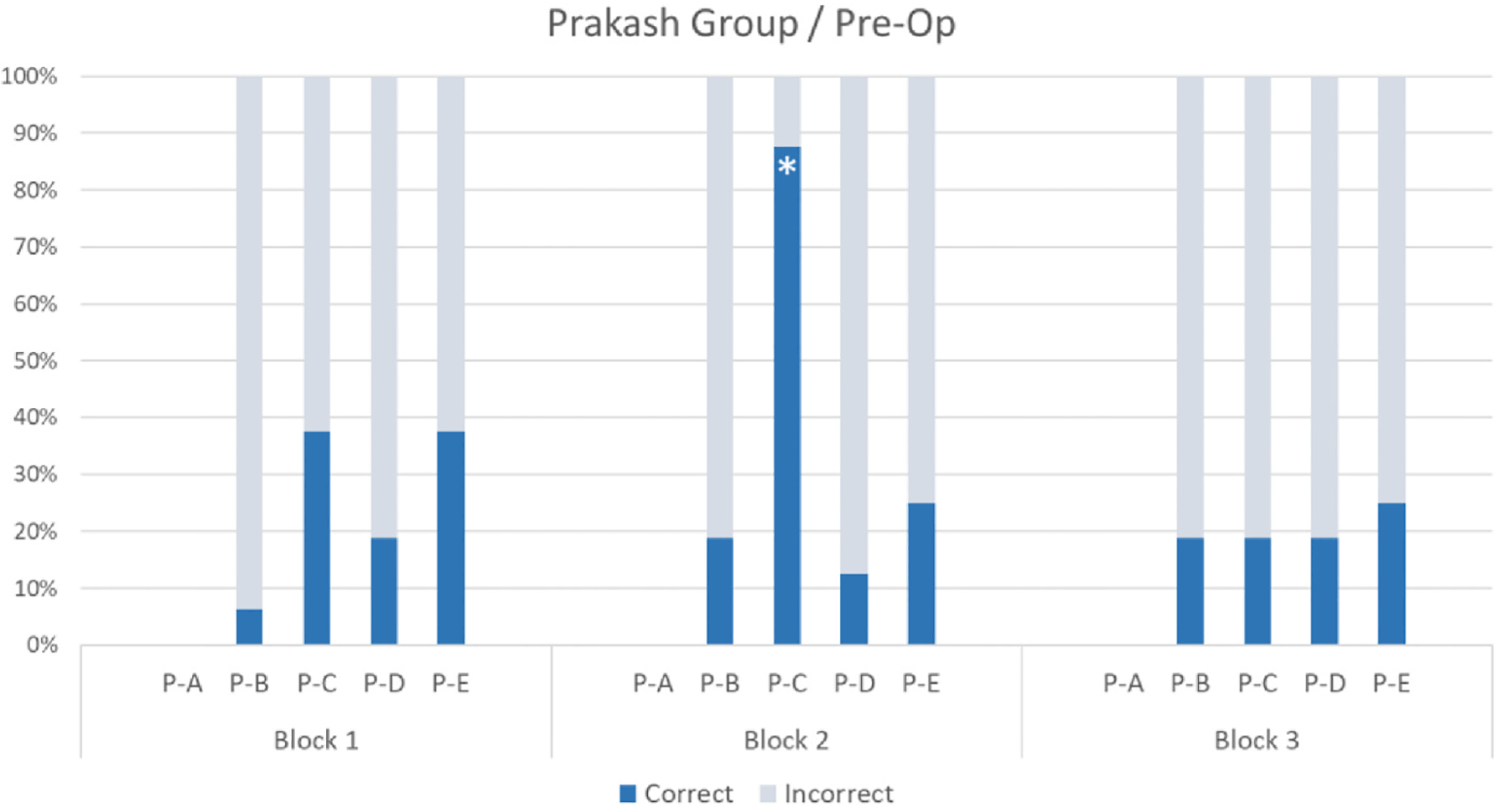
Mean percentages of correct and incorrect responses per participant and block (Block 1 = pre-intervention; Block 2 = intervention; Block 3 = post-intervention). Participant P-A could not perform the task pre-op because they did not have sufficient visual acuity to resolve the images on the screen. An asterisk indicates a significant difference relative to Block 1 (*p* < .01, Fisher’s Exact Test).

**Fig. 4.**
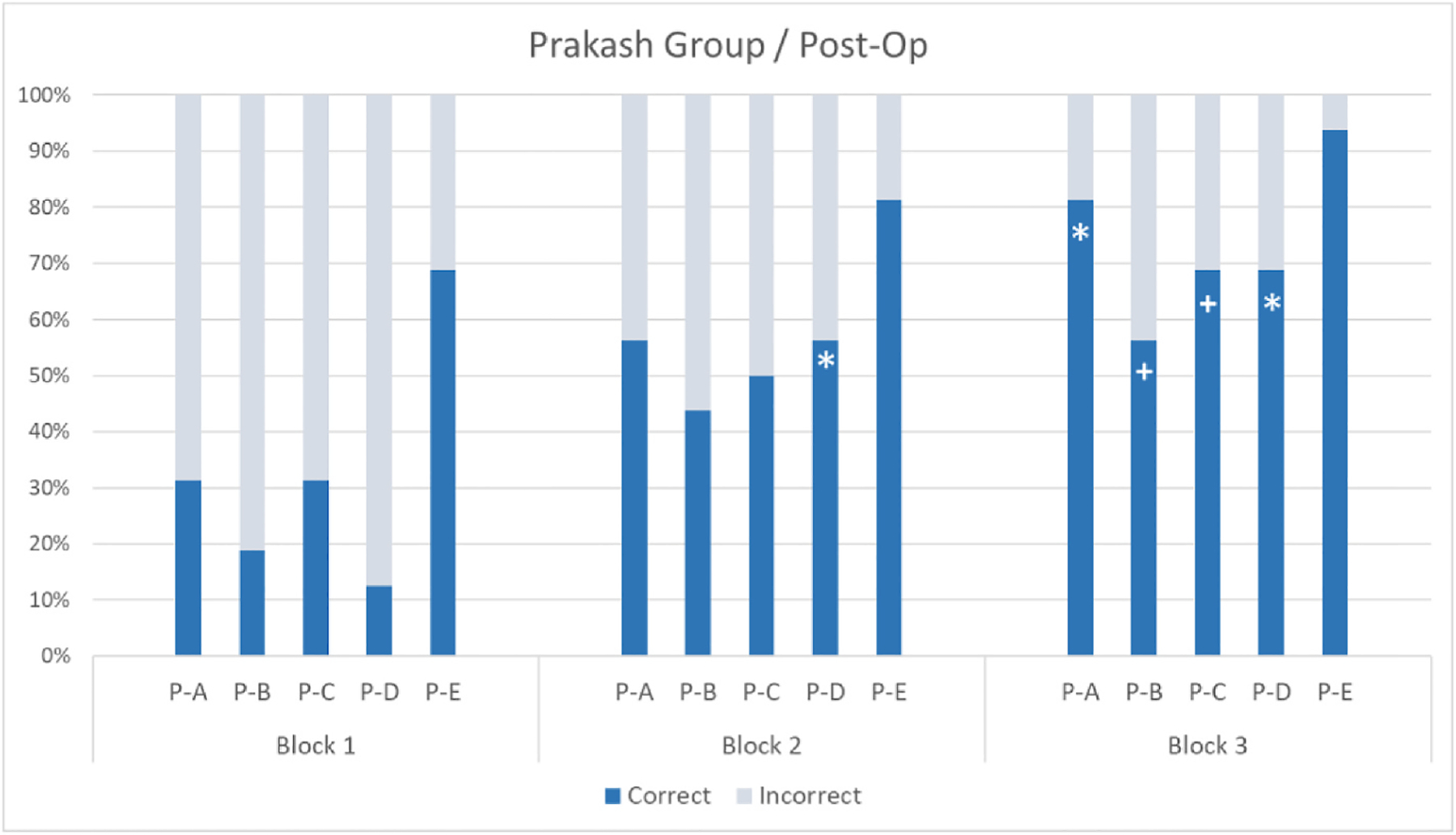
Mean percentages of correct and incorrect responses per participant and block (Block 1 = pre-intervention; Block 2 = intervention; Block 3 = post-intervention). Asterisk and cross indicate a significant difference (*p* < .024) and a marginally significant difference (*p* < .076) relative to Block 1, respectively (Fisher’s Exact Test).

Fisher’s Exact Tests were used to compare children’s correct and incorrect responses in Block 1 vs Block 2 (static vs dynamic cue) and Block 1 vs Block 3 (effect of intervening motion cues on static trials) at each test point. At the group level, there was no significant difference between Blocks 1 and 2 (*p* > .249) or between Blocks 1 and 3 (*p* > .673) before surgery. By contrast, the difference between Block 1 and Block 2 (*p* = .0024) and between Block 1 and Block 3 (*p* < .0001) were both significant one month after surgery. For individual performance, see Figs. 3 and 4.

These results suggest that prior to treatment, the Prakash group did not appreciate the motion cues in Block 2 (with the exception of participant P–C), while all participants benefitted from these cues after treatment. P–C’s performance pre-op suggests that he was more sensitive to motion cues compared to participants P–B, P-D and P-E prior to surgery, possibly because his poorer visual acuity left him unable to rely on static cues and therefore more dependent on motion cues. In addition, after the Prakash group received treatment, the motion cues in Block 2 facilitated their use of face orientation in static images in Block 3. It is worth noting the remarkable improvement of participant P-A, who could not perform the task prior to treatment due to their very poor visual acuity, while they responded correctly over 80% of the time in Block 3 after treatment.

#### 5.1.1. Silver Linings group

**Fig. 5.**
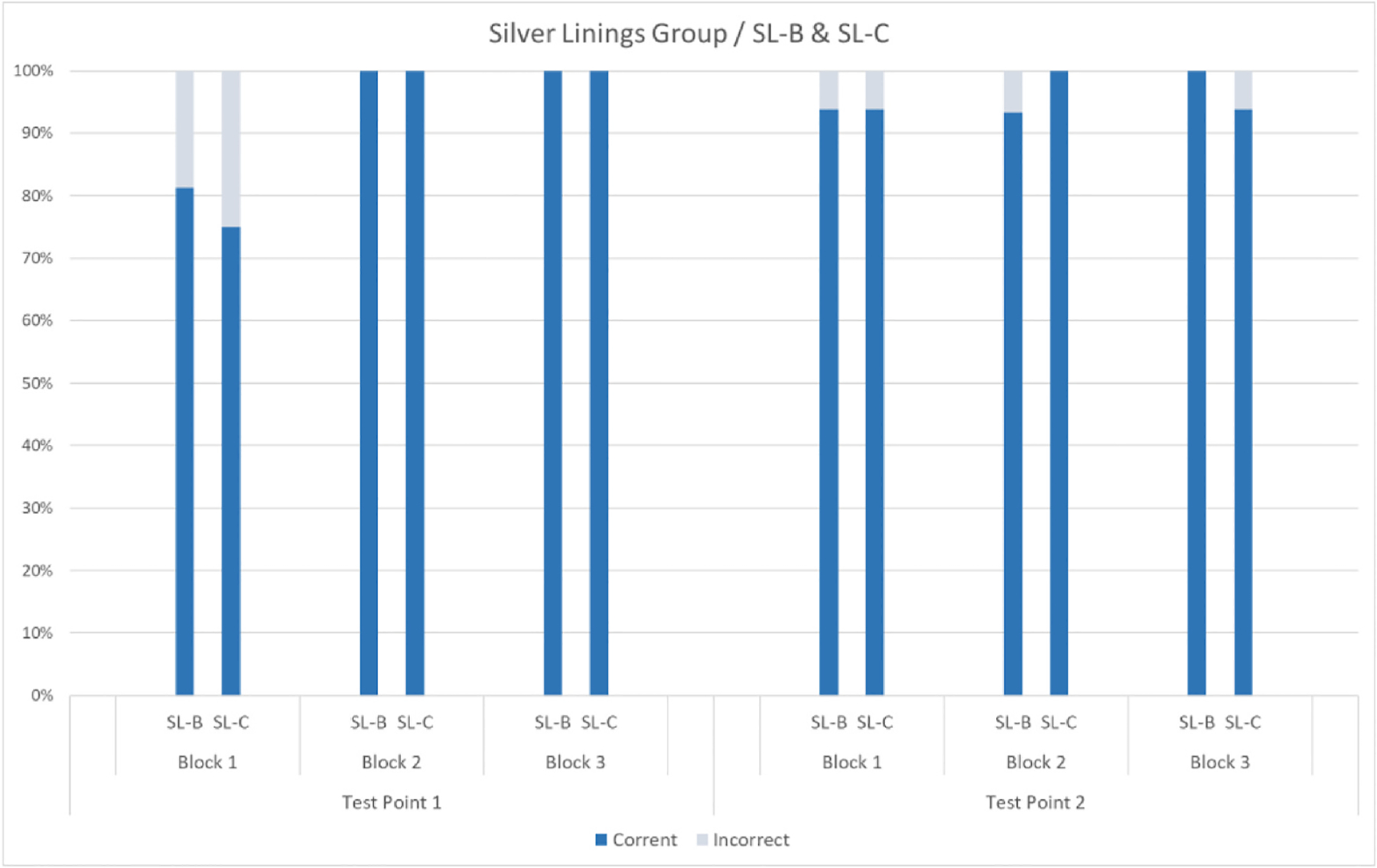
Mean percentages of correct and incorrect responses for participants SL-B and SL-C per block and test point (Block 1 = pre-intervention; Block 2 = intervention; Block 3 = post-intervention). There was a 6-month interval between Test Point 1 and Test Point 2.

Participants SL-B and SL-C were near ceiling from Block 1/Test Point 1 and continued to show high accuracy in their responses across blocks and testing sessions. For individual performance, see Fig. 5.

**Fig. 6.**
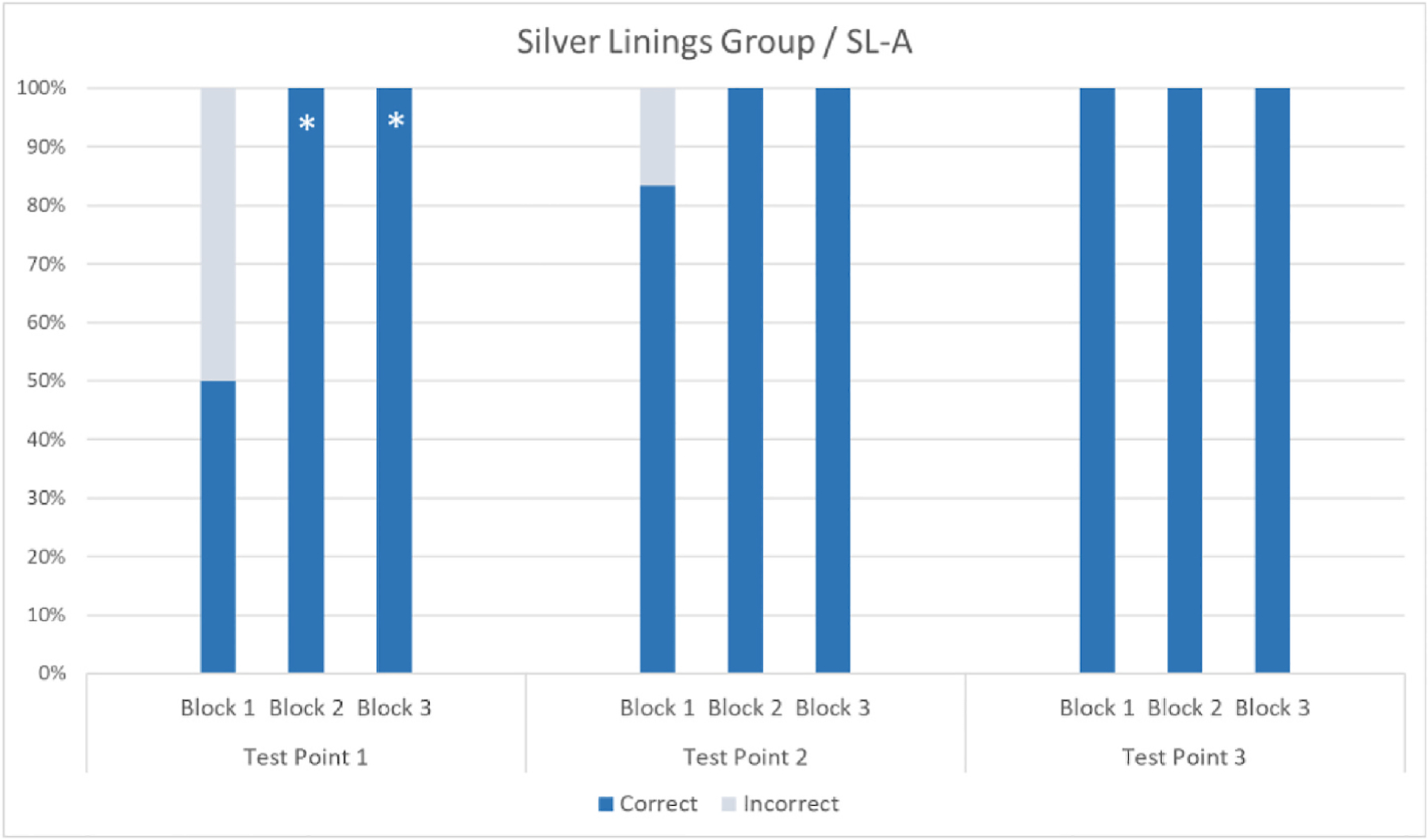
Mean percentages of correct and incorrect responses for participant SL-A per block and test point (Block 1 = pre-intervention; Block 2 = intervention; Block 3 = post-intervention). There was a 6-month interval between test points. Because of technical problems, only 12/16 and 14/16 responses were recorded in Block 1 and Block 2, respectively, at Test Point 2. Asterisks indicate a significant difference relative to Block 1 within the same testing session (*p* < .003, Fisher’s Exact Test).

Participant SL-A, who was younger and had lower visual acuity than SL-B and SL-C post-surgery, showed reliable improvement between Blocks 1 and 2 and between Blocks 1 and 3 at Test Point 1, and her improvement seemed to persist at Test Point 2 (see Fig. 6). However, because of connectivity issues in the second testing session, 25% and 12.5% of responses were not recorded in Block 1 and Block 2, respectively. One should therefore be cautious when comparing her performance across Test Points 1 and 2. However, SL-A showed ceiling performance in Block 1 at Test Point 3, confirming that her improvement was maintained a year later.

#### 5.1.2. Implicit learning across pre-intervention trials

While the performance of both Prakash and Silver Linings children suggests that they benefitted from the motion cues administered in Block 2, it is possible that they may have experienced implicit learning in Block 1. In other words, they may have improved through sheer practice, rather than in response to the motion cues. To address this question, we examined these children’s performance across the first block of trials (see Table 3). From the Silver Linings group, only Participant A was included in this analysis because Participants B and C were already near ceiling in Block 1/Test Point 1 (see Fig. 5).

Contrary to the implicit learning hypothesis, the results of a Fisher’s Exact Test revealed that this group of children performed better in the first half of Block 1 than in the second half (23 vs 11 correct responses), offering support to our interpretation of the main pattern of results as evidence of learning from motion cues.

#### 5.1.3. Control group

**Fig. 7.**
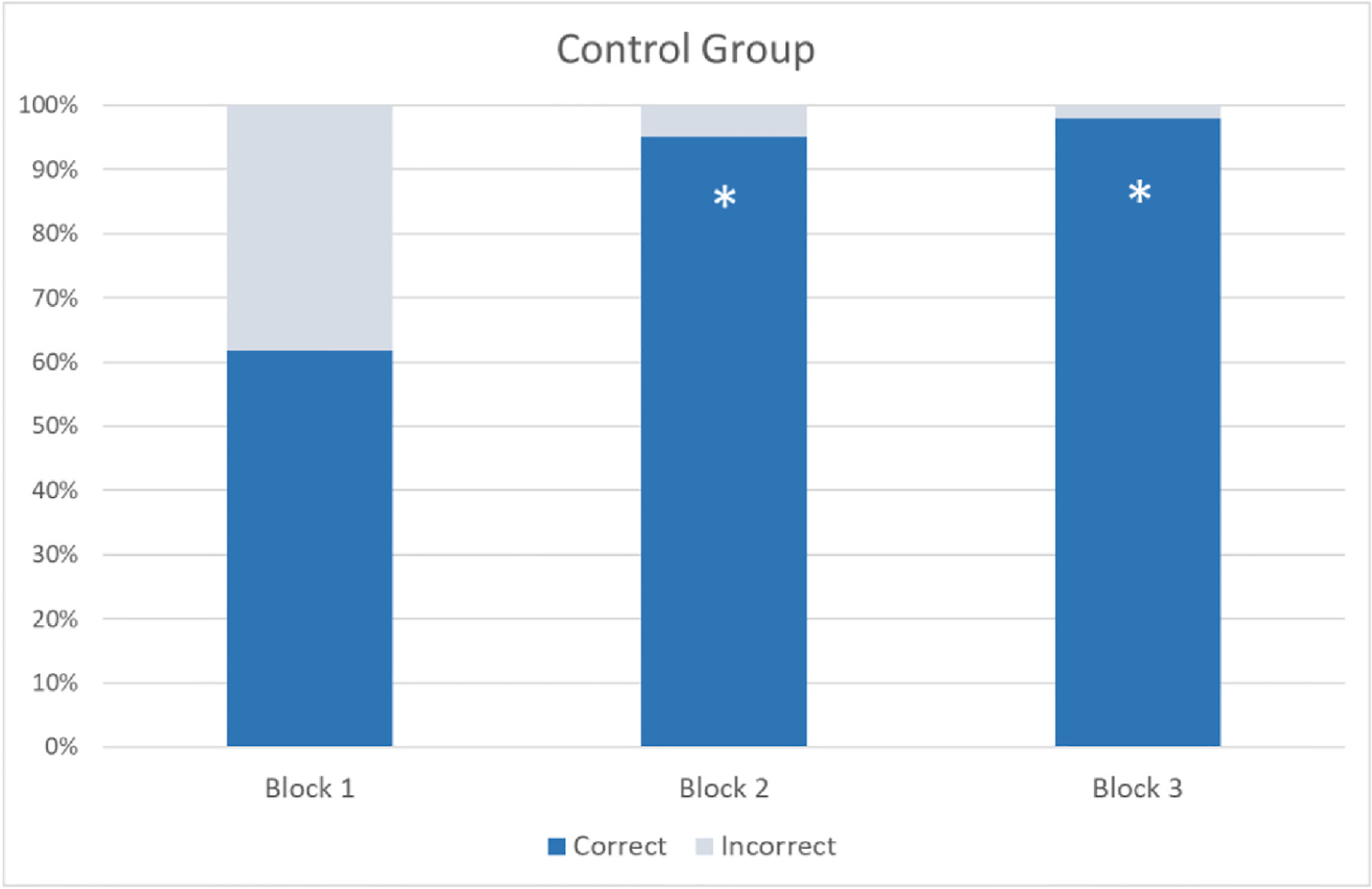
Mean percentages of correct and incorrect responses per block (Block 1 = pre-intervention; Block 2 = intervention; Block 3 = post-intervention). Asterisks indicate a significant difference relative to Block 1 (*p* < .0001, Fisher’s Exact Test).

The controls in Experiment 1 showed significant improvement between Blocks 1 and 2, and Blocks 1 and 3 (see Fig. 7). This pattern of results confirms that head turning is an effective cue for gaze following, not only for newly sighted children, but also for neurotypical children. In addition, like the Prakash and Silver Linings groups, the control group also used the motion cues provided in the second block of trials to interpret face orientation in static pictures in the last block.

**Fig. 8.**
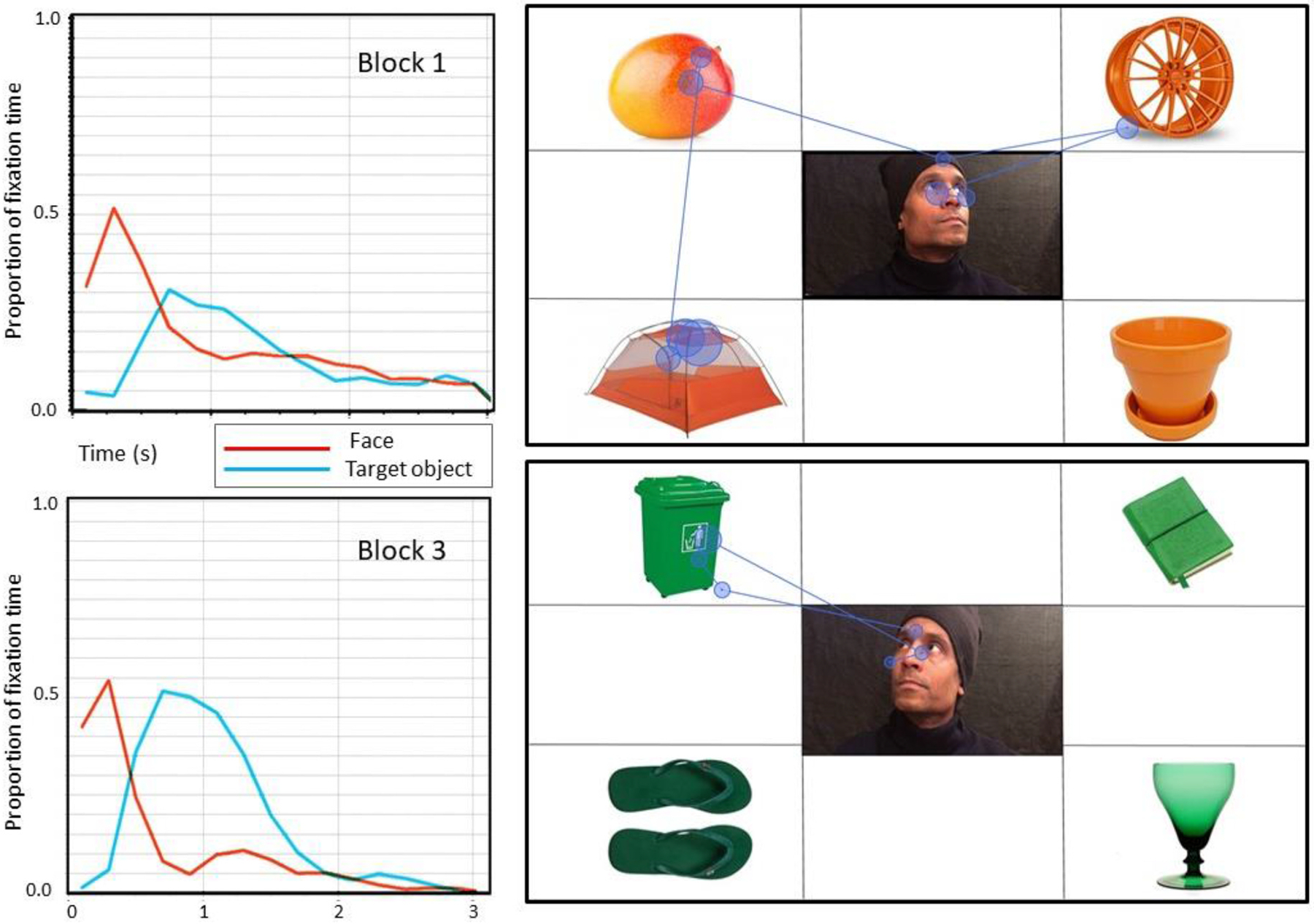
Mean proportions of fixation time on the man’s face and the target object during trials (trial fixed duration: 3 s) in Block 1 (pre-intervention; top left graph) and Block 3 (post-intervention; bottom left graph). The displays on the right-hand side show the scan path of one of the control children in Block 1 (top) and Block 3 (bottom).

Regarding children’s fixations on the man’s face and the target object across time, we observed a clear difference between the first and third trial blocks (see Fig. 8). In Block 1, the control children showed an initial preference for the man’s face but no comparable preference for the target object. By contrast, in Block 3, children looked first at the man’s face, before revealing a clear preference for the target object.

**Fig. 9.**
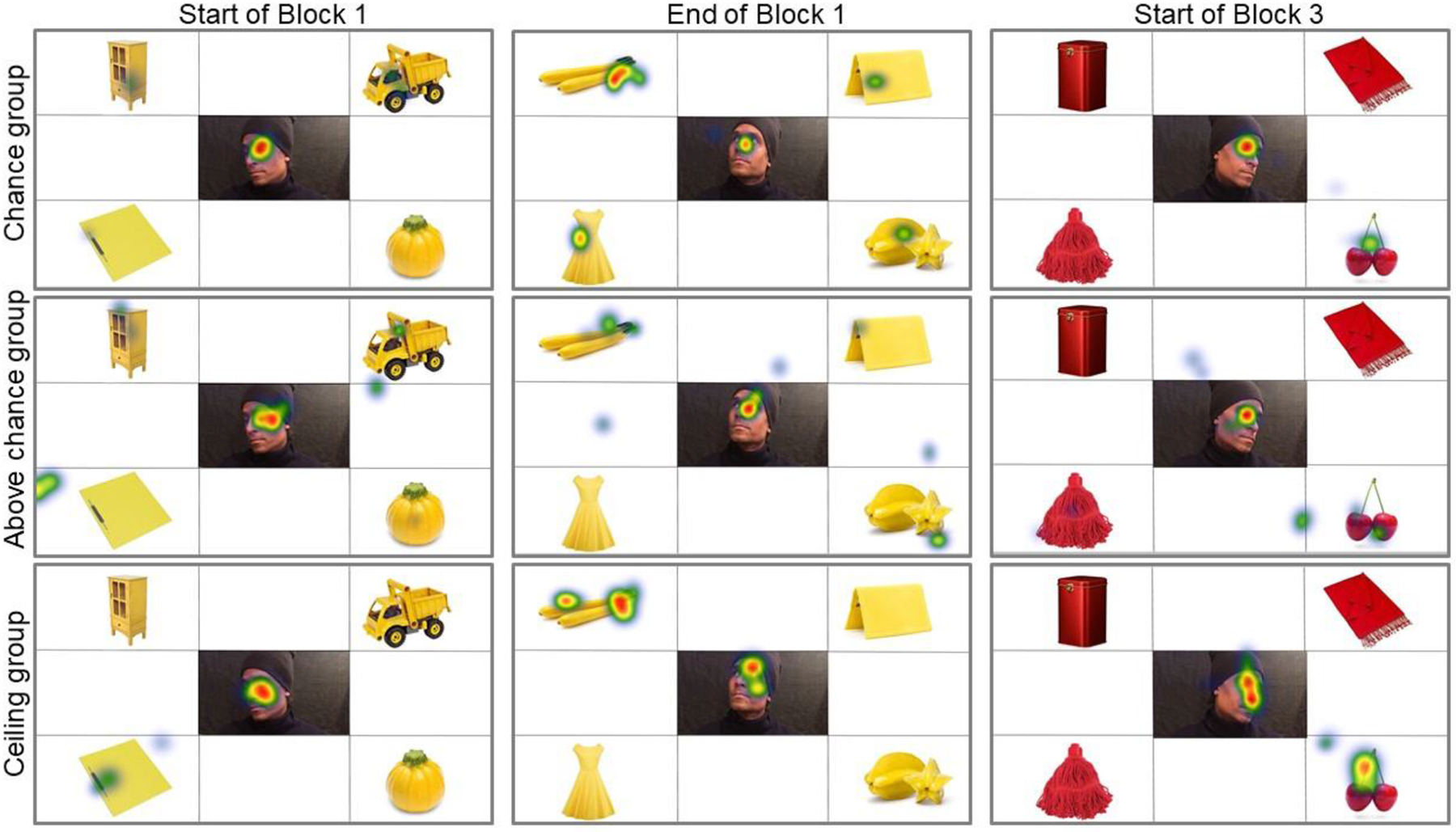
Distribution of fixations across three displays, corresponding with the first and last trials of Block 1 (static face condition/pre-intervention) and the first trial of Block 3 (static face condition/post-intervention). For this visualization, the control children in Experiment 1 were distributed in three groups according to their performance in Block 1 (3 children per group). The heatmap scale (indicating average fixation time) is the following: Blue/20–80 ms, Green/100–160 ms, Yellow/180–240 ms, and Red/260–320 ms.

Regarding individual performance, three control children were already at ceiling in Block 1 (15–16 correct/16 trials), another three were above chance (8–12 correct/16 trials) and the last three were at chance (3–5 correct/16 trials). Grouping these nine children by their performance in Block 1 revealed different patterns of fixations between Block 1 and Block 3 (see Fig. 9). In particular, unlike the children who were at ceiling from the start, those above chance and at chance were still considering all four objects in the display by the end of the first block. However, by the start of the third block, all children showed comparable looking behavior, with a clear preference for the man’s face and the target object. In conclusion, the control group’s performance in this task, both in terms of response accuracy and looking behavior, suggests that there is sufficient variability across blocks to extend the investigation to neurotypical children and adults. That was therefore the aim of Experiment 2.

## 6. Experiment 2

### 6.1. Methods

#### 6.1.1. Participants

Ninety children were recruited from a primary school in Delhi (India). The school serves middle-class families and teaches Kinder-garten to Grade 12 (ages 4–17). Arrangements were made with the School Principal so that the experimenters would contact the teachers in the participating grades, who would in turn contact the parents of the children and set up a 3-way Zoom call. Thirty children were recruited from Grade 1 (5–6 years), thirty from Grade 3 (7–8 years) and thirty from Grade 5 (age 9–10 years). In addition, a group of 30 students from Jawaharlal Nehru University and University of Delhi were contacted via email to volunteer in the study as adult controls. Sample size was determined by the time available for testing and previous developmental studies.

#### 6.1.2. Materials, task design and procedure

At the time of running Experiment 2, the COVID-19 pandemic was still ongoing. Therefore, the materials, task and procedure that had been used with the Silver Linings group in Experiment 1 were used again in Experiment 2 in order to administer the task online. The only difference were the practice trials, where pictures of everyday objects replaced the color patches that had been used with the Silver Linings group. The task was conducted again via Zoom. For the children, three-way calls were arranged, connecting a schoolteacher, the participating child (with a parent) and the experimenter. The adults were on a call with the experimenter.

## 7. Results

**Fig. 10.**
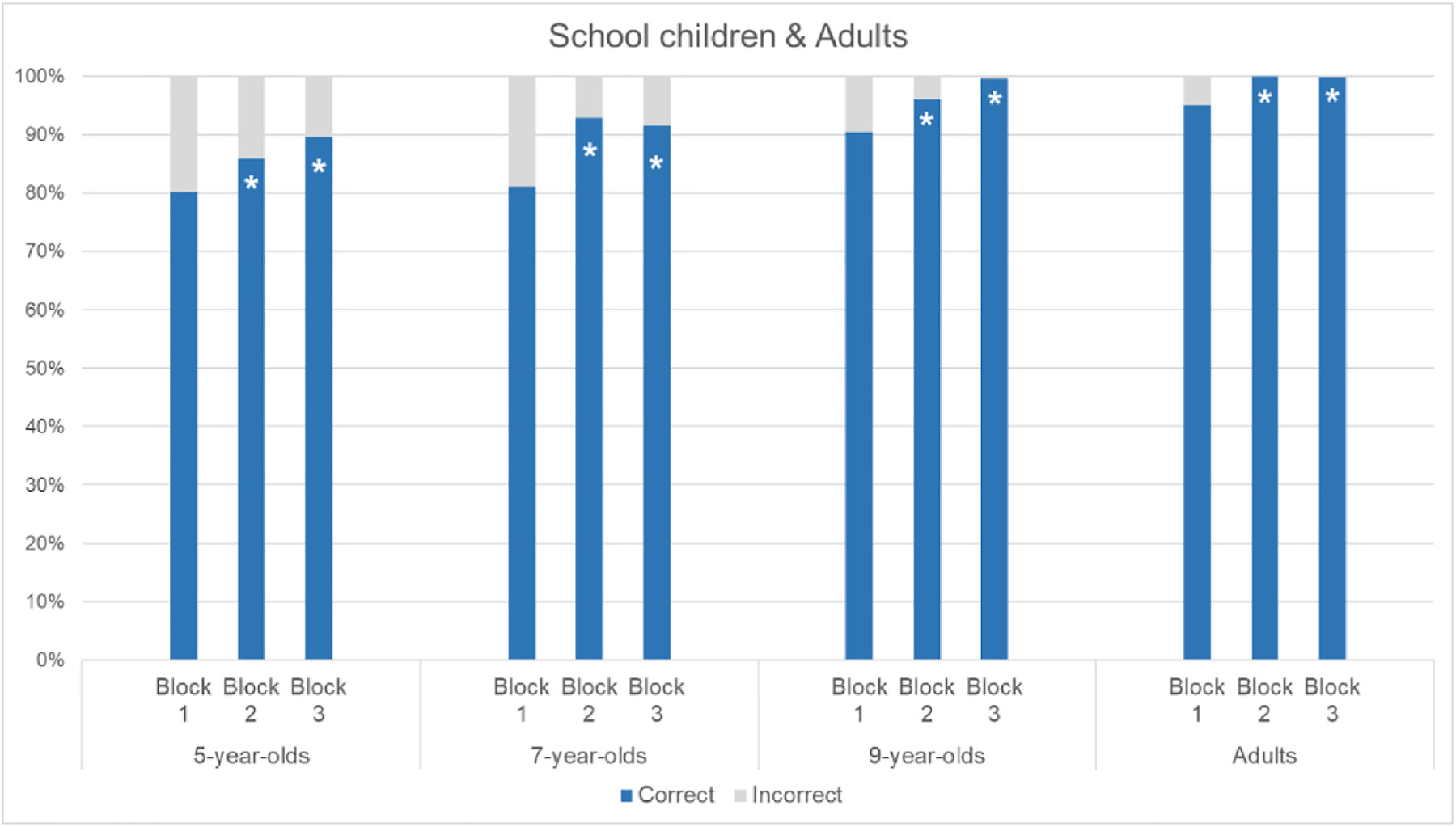
Mean percentages of correct and incorrect responses per age group and block (Block 1 = pre-intervention; Block 2 = intervention; Block 3 = post-intervention). Asterisks indicate a significant difference relative to Block 1 (*p* < .026, Fisher’s Exact Test).

All age groups in Experiment 2 revealed significant improvement between Blocks 1 and 2 (see Fig. 10), confirming the motion cues provided in the second block of trials made the task easier for both neurotypical children and adults (5-year-olds: *p* = .0252; 7-year-olds: *p* < .0001; 9-year-olds: *p* = .0007; adults: *p* < .0001; Fisher’s Exact tests). In addition, all age groups maintained their improvement across Blocks 1 and 3 (5-year-olds: *p* = .0001; 7-year-olds: *p* < .0001; 9-year-olds: *p* < .0001; adults: *p* < .0001; Fisher’s Exact tests).

### 7.1. Implicit learning across pre-intervention trials

While comparing participants’ performance across trial blocks revealed similar results in Experiment 1 and Experiment 2, an important difference emerged in the analysis of implicit learning: all age groups in Experiment 2 had started following the man’s gaze in the pre-intervention trials, prior to receiving the motion cues in the intervention trials. This was revealed by their improved accuracy in the second half of Block 1, which differed markedly from the Prakash children’s performance in Experiment 1 (see Table 3). These two patterns of results therefore suggest that whereas the Prakash children relied on motion cues for gaze following, our neurotypical participants only benefitted from those motion cues – but did not depend on them. Developmental trends in Experiment 2 can be observed both in the number of participants who performed at ceiling and at chance across trial blocks (see Table 4).

## 8. General discussion

Gaze following (the ability to look where someone else is looking) has been extensively investigated as an early social skill that can even be observed in newborns (Farroni et al., 2002, 2005). This ability is particularly important for word learning because it gives young children a cue to map new words onto the corresponding referents (Brooks and Meltzoff, 2005). Here we investigated, for the first time, whether newly sighted children (who were treated for dense congenital cataracts after several years of visual deprivation) rely on head turning as a cue for gaze following in referential communication, and to what extent head motion cues generalize to static face orientation (Experiment 1). The investigation was also extended to neurotypical school children (ages 5–10 years) and adults (Experiment 2).

Using a block design with pre-intervention, intervention, and post-intervention trials, we showed that head motion is an effective cue for gaze following in referential communication, with the three groups tested in the study benefitting from the head motion cues. The Prakash children in Experiment 1 showed a transfer effect from appreciating the referential intent of head motion in the intervention trials to the interpretation of static face orientation in post-intervention trials. In addition, the Prakash group revealed remarkable improvement one month after surgery, while the Silver Linings group maintained their improvement across testing sessions at 6-month intervals. Our results therefore extend previous work on the importance of motion cues for individuals with late sight onset (Ostrovsky et al., 2006, 2009; Gilad-Gutnick et al., 2019), confirming that head turning facilitates their referential communication.

Unlike the Prakash children in our sample, the neurotypical children and adults in Experiment 2 significantly improved their accuracy in target selection within the first block of trials (pre-intervention), confirming that, while they benefit from motion cues, they do not rely on head turning as a cue for gaze following. Given that participants in Experiment 2 were able to reliably use gaze direction in static images as a cue for referential intent, one may wonder why they did not reveal ceiling performance from the start of the task. Based on adult participants’ feedback during debriefing, we hypothesize that some participants were initially trying to guess the meaning of the foreign words they heard, rather than focusing on the direction of the man’s gaze. However, this explanation may not apply to participants of all ages, making it compatible with developmental trends in our sample.

### 8.1. Developmental pathways to joint attention

Experimental research with infants has long established developmental connections between sensorimotor development, visual object recognition and word learning (Thelen and Smith, 1994, 2007; Smith et al., 2018), supporting a ‘pathways approach’ to human development and evolution (Smith, 2013). For example, the ability to seat steadily and manipulate objects is part of the developmental pathway leading to visual object recognition, which is in turn fundamental to word learning. Smith (2013) identifies two ways in which developmental pathways to specific outcomes are complex: they are multicausal (i.e. each change is dependent on multiple causes) and also degenerate (i.e. there is more than one route to the same functional end). In children with congenital cataracts, the reliance on head movement as a cue for gaze following can be understood as an alternative route to joint attention, in tandem with their sensitivity to voice direction.

However, not all aspects of face perception can be compensated after an extended period of blindness. Early experience with faces seems to be crucial for the development of *configural face processing* (i.e. the sensitivity to the spacing among facial features). Individuals with congenital cataracts who were treated as early as 4 months after birth have shown permanent deficits in configural face processing, even though this is a late-emerging visual skill that is not fully mature until adolescence (Maurer et al., 2007; Maurer, 2017). Similarly, recent work using event related potentials (ERPs) with individuals who had been treated for congenital cataracts between 2 months and 14 years of age support the view that the functional specialization of the neural system for human face processing is dependent on early experience and linked to a sensitive period (Röder et al., 2013). However, in a more recent study, Gandhi et al. (2017) observed that newly sighted individuals were unable to distinguish between faces and nonfaces immediately after sight onset, but showed remarkable improvement in the following months. In view of these results, the authors highlight these population’s preserved plasticity for acquiring face/non-face categorization even late in life. Zerr et al. (2020) also showed that congenital cataract reversal individuals were able to perform visually guided gaze shifts, even when their blindness had lasted for decades (with measures of latency and accuracy comparable to a nystagmus control group).

Given the accumulating evidence of preserved plasticity for visual development in individuals with late visual onset (Ostrovsky et al., 2006, 2009; Gandhi et al., 2017; Zerr et al., 2020), and the general observation that visual attention is normally biased in the direction of perceived movement (Smith, 2013), it seems safe to assume that head motion offers Prakash children an alternative developmental pathway for gaze following in communicative settings. In this view, accurate detection of pupil direction is not necessary to follow someone’s line of gaze: head turning can be a reliable proxy.

Forthcoming work by Zohary et al. (2022) shows that the visual acuity of newly sighted individuals typically improves substantially after surgery, allowing discrimination of direction of pupil position in the eye. However, these patients failed to show gaze cueing effects and fixated less on the eyes than their neurotypical controls. Zohary et al.’s computational model of unsupervised learning of gaze-direction explains how head-based gaze following can develop under severe image blur (resembling pre-surgery conditions in patients with congenital bilateral cataracts). The model also suggests that lack of detailed early visual experience hinders automatic gaze following in late visual onset individuals, even if they gain sufficient resolution to extract eye position after treatment.

We interpret the results of our word-learning study in line with Zohary et al.’s (2022) findings: for people with late visual onset, detecting an interlocutor’s head turn is an important cue to establish joint attention, above and beyond static gaze direction.

### 8.2. Strong directionality cues can be strong intentionality indicators

An important question that the present results do not address is the degree to which following a head turn depends on (or benefits from) first establishing eye contact. The videos in this study started with the man looking ahead, in a neutral position, which may have resulted in eye contact with the participants. Developmental studies have shown that eye contact is crucial for infants to recognize ostensive cues and communicative intent (Okumura et al., 2013a, 2013b, 2020). These findings suggests that the man’s looking ahead in our videos could have engaged participants in following his head turning towards the target object. Future studies should investigate this possibility, since it would help us better understand the degree to which head following is a reflex response (triggered by a general bias to follow perceived motion direction; Smith, 2013), or a social cue (that is affected by the presence or absence of initial eye contact; Brooks and Meltzoff, 2002; Meltzoff and Brooks, 2007; Csibra and Gergely, 2009).

In line with the developmental literature, here we treated head motion as a social cue because we were interested in the role of head motion in word learning, which is a fundamentally social activity (Baldwin and Moses, 2001; Brooks and Meltzoff, 2005). However, we acknowledge that non-social cues, such as moving arrows, may allow children to map words onto their physical referents in laboratory tasks (for recent evidence with neurotypical children, see Bang and Nadig, 2020). We would in fact predict that as long as a non-social motion cue is strong enough, Prakash children should be able to shift their attention in the intended direction and potentially map new words onto their referents. However, children do not learn words from moving arrows in their everyday lives, and in that respect, investigating Prakash children’s use of non-social motion cues for word learning is beyond the scope of this study. Future experiments should try to determine whether late visual onset individuals are more or less sensitive to motion cues depending on their social or non-social nature.

From a perceptual point of view, head turning is a stronger directionality cue than face orientation or gaze direction in static images (more so even than the pupil shifts used in infant studies; see Farroni et al., 2000, 2003). Visual salience alone could therefore explain why head turning is such an effective gaze-following cue for Prakash children. However, from a Theory of Mind perspective, the benefit of head turning may go beyond perception: as a social cue, head turning is a more reliable indicator of referential intent than static gaze direction. After all, one may look at something for a number of reasons, without necessarily revealing referential intent (e.g., we may stare in a certain direction while thinking of something else, or even look at one thing while talking about another). However, directed head turning unequivocally signals a shift in someone’s visual attention, working as a more reliable cue for word learning.

In addition to signaling shifts in others’ visual attention, head turning correlates with voice direction and a number of adult behaviors that may further support Prakash children’s word learning (e.g., pointing, reaching or manipulating objects). In this view, motion not only of the head, but also of the hands (in instances of touching and pointing) provides effective directionality cues that support word learning. Developmental work with neurotypical infants has revealed that babies often shift their gaze from an adult’s face to an adult’s hand, which has been interpreted as a precursor of joint attention (Amano et al., 2004). Computational models have also shown that agent motion events provide an internal teaching signal that allows infants to acquire hand and gaze representations (Ullman et al., 2012), which can support word learning later in development.

In conclusion, head turning is a strong directionality cue that reliably conveys information about others’ attentional states and correlates with their voice direction and hand motion, thus playing a fundamental role in children’s word learning and socio-cognitive development.

## Figures and Tables

**Table 1:** Demographic and clinical information for the Prakash group.

Participant	Age at test	Date of surgery	Type of cataract	PreOp acuity (in logMAR)	PostOp Accuity (in logMAR)
P-A	20	Jan-20	Total	2.49	1mo: 2.13
P-B	13	Jan-20	Total	2.05	1mo: 2.11
P-C	15	Jan-20	Total	2.44	1mo: 2.13
P-D	7	Jan-20	Total	1.52	1mo: 0.95
P-E	13	Jan-20	Total	1.44	1mo: 1.44

**Table 2:** Demographic and clinical information for the Silver Linings group.

Participant	Age at surgery	Date of surgery	Type of cataract	Age at test	PreOp acuity (in logMAR)	PostOp Accuity (in logMAR)
SL-A	7	Jan-18	Nuclear	10	1.87	12mo: 1.42
SL-B	11	Dec-12	Membranous	18	FC@30 cm	8 y: 1.12
SL-C	12	Jan-19	Membranous	13	1.53	6mo: 1.04

Note: FC = Finger Counting.

**Table 3:** Individual performance (1 = correct response) across Block 1 (Trials 1–16) for the Prakash group (Post-Op) and Silver Linings girl A (Test Point 1).

	1	2	3	4	5	6	7	8	9	10	11	12	13	14	15	16
**P-A**	0	0	0	0	1	0	1	1	0	0	0	1	0	1	0	0
**P-B**	0	0	1	1	0	0	0	1	0	0	0	0	0	0	0	0
**P-C**	1	1	1	0	0	1	0	1	0	0	0	0	0	0	0	0
**P-D**	0	0	0	0	0	0	0	1	0	0	0	0	1	0	0	0
**P-E**	1	1	1	1	1	0	0	1	1	1	1	0	1	0	1	0
**SL-A**	1	1	1	1	0	0	0	1	1	0	1	0	1	0	0	0

**Table 4:** Number (and percentage) of children at ceiling and chance levels across blocks (Block 1 = pre-intervention; Block 2 = intervention; Block 3 = post-intervention).

	5-year-olds	7-year-olds	9-year-olds	Adults
At ceiling in Block 1	7 (23%)	7 (23%)	14 (47%)	17 (57%)
At ceiling in the 2nd half of Block 1	24 (80%)	24 (80%)	26 (87%)	29 (97%)
Chance or below in Block 1	5 (17%)	6 (20%)	1 (3%)	0 (0%)
Chance or below in Block 2	5 (17%)	3 (10%)	1 (3%)	0 (0%)
Chance or below in Block 3	4 (13%)	3 (10%)	0 (0%)	0 (0%)

## Footnotes

1 All Prakash children had significant nystagmus due to the extended visual deprivation they had suffered. This rendered eye-tracker calibration very difficult.
